# Hexakis(2-amino-4-methyl­pyridine-κ*N* ^1^)dioxidohexa-μ_4_-sulfido-hexa­copper(I)divanadium(V)

**DOI:** 10.1107/S1600536808027165

**Published:** 2008-08-30

**Authors:** Zhang Yu, Guodong Tang, Jianying Zhao, Zhengjing Jiang

**Affiliations:** a Department of Chemistry, Huaiyin Teachers College, Huai’an 223300, Jiangsu, People’s Republic of China

## Abstract

The title compound, [Cu_6_V_2_O_2_S_6_(C_6_H_8_N_2_)_6_], is constructed from six CuS_3_N and two VOS_3_ distorted tetra­hedra, forming an octa­nuclear V/S/Cu cluster with *C*_*i*_ symmetry. The geometry around the V atoms is slightly distorted tetra­hedral, while there are large distortions from ideal tetra­hedral geometry for the Cu atoms. Adjacent metal–metal distances range from 2.693 (1) to 2.772 (10) Å, indicating weak metal–metal inter­actions in the cluster.

## Related literature

The most relevant known analog of the title compound is hexa­kis(μ_4_-sulfido)-dioxohexa­kis(triphenyl­phosphine) -hexa­copper(I)divanadium(V) (Zheng *et al.*, 2001 ▶), For related literature, see: Du *et al.* (1992 ▶); Holm (1992 ▶); Hou *et al.* (1996 ▶); Liu *et al.* (1995 ▶); Naruta *et al.* (1994 ▶); Zhang *et al.* (1996 ▶, 2001 ▶).
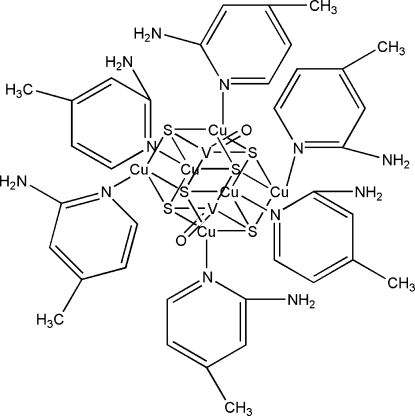


## Experimental

### 

#### Crystal data

[Cu_6_V_2_O_2_S_6_(C_6_H_8_N_2_)_6_]
*M* *_r_* = 1356.34
Hexagonal, 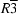

*a* = 14.139 (2) Å
*c* = 20.830 (4) Å
*V* = 3606.2 (10) Å^3^
*Z* = 3
Mo *K*α radiation
μ = 3.28 mm^−1^
*T* = 293 (2) K
0.3 × 0.2 × 0.15 mm

#### Data collection

Bruker APEXII CCD diffractometer
Absorption correction: multi-scan (*SADABS*; Bruker, 2000 ▶) *T*_min_ = 0.465, *T*_max_ = 0.611
6168 measured reflections
1837 independent reflections
1092 reflections with *I* > 2σ(*I*)
*R*_int_ = 0.054

#### Refinement

*R*[*F* ^2^ > 2σ(*F* ^2^)] = 0.050
*wR*(*F* ^2^) = 0.128
*S* = 1.02
1837 reflections
97 parameters
H-atom parameters constrained
Δρ_max_ = 0.58 e Å^−3^
Δρ_min_ = −0.69 e Å^−3^

### 

Data collection: *APEX2* (Bruker, 2004 ▶); cell refinement: *SAINT* (Bruker, 2004 ▶); data reduction: *SAINT*; program(s) used to solve structure: *SHELXS97* (Sheldrick, 2008 ▶); program(s) used to refine structure: *SHELXL97* (Sheldrick, 2008 ▶); molecular graphics: *ORTEP-3 for Windows* (Farrugia, 1997 ▶); software used to prepare material for publication: *SHELXL97* and *PLATON* (Spek, 2003 ▶).

## Supplementary Material

Crystal structure: contains datablocks I, global. DOI: 10.1107/S1600536808027165/bv2103sup1.cif

Structure factors: contains datablocks I. DOI: 10.1107/S1600536808027165/bv2103Isup2.hkl

Additional supplementary materials:  crystallographic information; 3D view; checkCIF report

## Figures and Tables

**Fig. 1.**
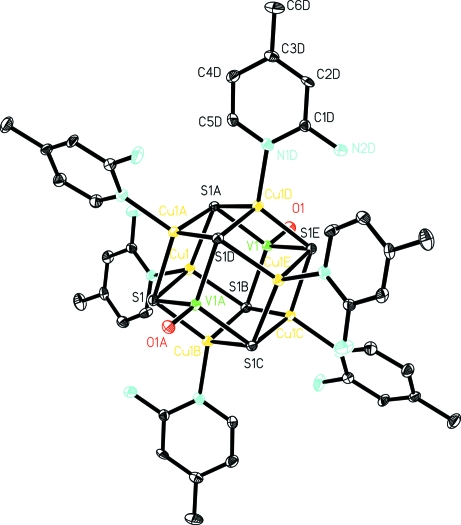
The molecular structure of (I), with atom labels and 30% probability displacement ellipsoids. All H atoms have been omitted.

**Fig. 2.**
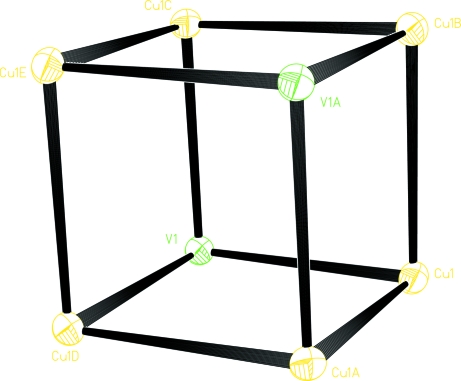
Cubic arrangement of metal atoms.

## supplementary crystallographic information

### Comment

In the past two decades, considerable attention has been directed to the study of tetrathiometalate anions [MXS_3_]^n-^ (*X*=O, S; *M*=V, Mo, W, Re) cluster compounds, since these complexes play a special role in catalysis reactions (Du *et al.*, 1992), biological processes (Holm *et al.*, 1992) and advanced materials (Naruta *et al.*, 1994). These moieties can react as multidentate ligands with a wide variety of metal ions, such as Cu, Ag, Au, Zn, Cd, Hg, Fe, Co, Ni, Pd, Pt, Sn, and Ru to form a wide range of novel structures (Hou *et al.*, 1996). More than 300 heterothiometallic cluster compounds containing these moieties have been synthesized and extensively studied (Zhang *et al.,*, 2001). However, crystal structures of these clusters containing 2-amino-4-methylpyridine ligands have not been reported until now.

In order to explore the chemistry of Mo(W)/S/Cu(Ag) clusters extensively, we have synthesized such a cluster by reaction in solution at normal temperatures. The solid-state molecular structure of the octanuclear neutral cluster 1 is shown in Fig. 1. It contains a cluster core [V_2_Cu_6_S_6_O_2_], of which the V_2_Cu_6_ atoms form a distorted cube, shown in Fig. 2. Each µ_4_-S atom is bonded to three Cu atoms and one V atom constructing each face of the dodecahedron. The geometry around the V atoms is slightly distorted tetrahedral with S–V–S 109.97 (5)° and S–V–O 108.97 (5)°, and the V–S bonds, 2.2382 (15) Å, are somewhat longer than those of the free [VS_4_]^3-^ anion as expected [2.17 Å in the ammonium salt]. The coordination geometry of every Cu atom, bonded to three µ_4_-S atoms and one terminal ligand 2-amino-4-methylpyridine, is strongly distorted from an ideal tetrahedron with S—Cu—N angles varying from 104.52 (13)° to 121.11 (14)°. This phenomenon may arise from the steric effect of the bulky 2-amino-4-methylpyridine ligands. The Cu—N distance of 2.033 (4) Å is somewhat longer than the Cu—N distance found in [V_2_S_4_O_3_(CuPPh_3_)_4_(CuMeCN)_2_] complexes (Zhang *et al.,*, 1996). The Cu—S distances between 2.2886 (15) Å and 2.4701 (16) Å are comparable to those reported in (Et_4_N)_3_[(VS_4_Cu_4_(Et_2_dtc)(PhS)_3_] (Et_2_dtc=diethyldithiocarbamate) complexes (mean Cu—S = 2.236 (5) Å)(Liu*et al.*, 1995).

In the preparation of the title compound, one S atom of the [VS_4_]^3-^ unit is replaced by an O atom and [VS_4_]^3-^ becomes [VS_3_O]^3-^. The V—O distance 1.618 (6) Å is a typical double bond distance. The adjacent metal-metal distances range from 2.6932 (11) Å to 2.7725 (10) Å, and are slightly shorter than normal V—Cu and Cu—Cu distances, indicating that there are weak metal-metal interactions. The terminal 2-amino-4-methylpyridine ligand is present in the usual monodentate mode. The C1—N1, C5—N1 and C1—N2 distances of 1.344 (6) Å, 1.344 (7) Å and 1.350 (7) Å, respectively, are typical C*sp*^2^—Nsp^2^ values.

### Experimental

To a solution of 2-amino-4-methylpyridine (0.0230 g, 0.1 mmol) in dimethylformamide (DMF) (10 ml) were added a solution of CuI (0.0741 g, 0.2 mmol) and (NH_4_)_3_VS_4_ suspended in DMF (5 ml). The reaction mixture was stirred at room temperature for about 8 h. The deep brown solution was filtered and slow diffution of *i*-PrOH/MeCN to the solution, resulted in black prismatic crystals suitable for X-ray analysis.

### Refinement

The amino hydrogen atoms were found from Fourier difference maps and fixed with N—H bond lengths of 0.90 Å. The H atoms of the aromatic group were geometrically idealized. All the H atoms were refined isotropically with isotropic vibration parameters related to the atoms to which they are bonded with *U*ĩso~ = 1.2 *U*~eq~ (*U*ĩso~ = 1.5*U*~eq~ for methyl H atoms).

### Crystal data

**Table:**

[Cu_6_V_2_O_2_S_6_(C_6_H_8_N_2_)_6_]	*Z* = 3
*M**_r_* = 1356.34	*F*_000_ = 2040
Hexagonal, *R*3	*D*_x_ = 1.874 Mg m^−^^3^
Hall symbol: -R 3	Mo *K*α radiation λ = 0.71073 Å
*a* = 14.139 (2) Å	Cell parameters from 6634 reflections
*b* = 14.139 (2) Å	θ = 1.9–27.5º
*c* = 20.830 (4) Å	µ = 3.28 mm^−^^1^
α = 90º	*T* = 293 (2) K
β = 90º	Block, black
γ = 120º	0.3 × 0.2 × 0.15 mm
*V* = 3606.2 (10) Å^3^	

### Data collection

**Table:**

Bruker APEXII CCD diffractometer	1837 independent reflections
Radiation source: fine-focus sealed tube	1092 reflections with *I* > 2σ(*I*)
Monochromator: graphite	*R*_int_ = 0.054
*T* = 293(2) K	θ_max_ = 27.5º
φ and ω scans	θ_min_ = 1.9º
Absorption correction: multi-scan(SADABS; Bruker, 2000)	*h* = −17→18
*T*_min_ = 0.465, *T*_max_ = 0.611	*k* = −12→18
6168 measured reflections	*l* = −26→27

### Refinement

**Table:**

Refinement on *F*^2^	Secondary atom site location: difference Fourier map
Least-squares matrix: full	Hydrogen site location: inferred from neighbouring sites
*R*[*F*^2^ > 2σ(*F*^2^)] = 0.050	H-atom parameters constrained
*wR*(*F*^2^) = 0.128	*w* = 1/[σ^2^(*F*_o_^2^) + (0.0525*P*)^2^] where *P* = (*F*_o_^2^ + 2*F*_c_^2^)/3
*S* = 1.02	(Δ/σ)_max_ = 0.001
1837 reflections	Δρ_max_ = 0.58 e Å^−^^3^
97 parameters	Δρ_min_ = −0.69 e Å^−^^3^
Primary atom site location: structure-invariant direct methods	Extinction correction: none

### Special details

**Table:**

Geometry. All e.s.d.'s (except the e.s.d. in the dihedral angle between two l.s. planes) are estimated using the full covariance matrix. The cell e.s.d.'s are taken into account individually in the estimation of e.s.d.'s in distances, angles and torsion angles; correlations between e.s.d.'s in cell parameters are only used when they are defined by crystal symmetry. An approximate (isotropic) treatment of cell e.s.d.'s is used for estimating e.s.d.'s involving l.s. planes.
Refinement. Refinement of *F*^2^ against ALL reflections. The weighted *R*-factor *wR* and goodness of fit *S* are based on *F*^2^, conventional *R*-factors *R* are based on *F*, with *F* set to zero for negative *F*^2^. The threshold expression of *F*^2^ > σ(*F*^2^) is used only for calculating *R*-factors(gt) *etc*. and is not relevant to the choice of reflections for refinement. *R*-factors based on *F*^2^ are statistically about twice as large as those based on *F*, and *R*- factors based on ALL data will be even larger.

### Fractional atomic coordinates and isotropic or equivalent isotropic displacement parameters (Å^2^)

**Table:**

	*x*	*y*	*z*	*U*_iso_*/*U*_eq_	
Cu1	0.15031 (5)	0.98780 (5)	0.96002 (3)	0.0233 (3)	
V1	0.0000	1.0000	0.88657 (7)	0.0207 (4)	
S1	0.14481 (11)	0.99064 (11)	1.07850 (6)	0.0200 (4)	
N1	0.2966 (4)	1.0004 (4)	0.9392 (2)	0.0247 (12)	
N2	0.2279 (4)	0.8233 (4)	0.9034 (2)	0.0412 (14)	
H2A	0.1631	0.8107	0.9120	0.049*	
H2B	0.2373	0.7723	0.8878	0.049*	
C2	0.4198 (5)	0.9427 (5)	0.9031 (3)	0.0269 (14)	
H2C	0.4295	0.8870	0.8865	0.032*	
O1	0.0000	1.0000	0.8089 (3)	0.0252 (16)	
C1	0.3149 (5)	0.9230 (5)	0.9147 (2)	0.0227 (13)	
C4	0.4909 (5)	1.1207 (5)	0.9417 (3)	0.0309 (15)	
H4A	0.5492	1.1893	0.9518	0.037*	
C5	0.3854 (5)	1.0969 (5)	0.9528 (3)	0.0296 (15)	
H5A	0.3749	1.1511	0.9710	0.036*	
C3	0.5092 (5)	1.0422 (5)	0.9155 (3)	0.0255 (14)	
C6	0.6221 (5)	1.0633 (6)	0.9020 (3)	0.0424 (18)	
H6A	0.6744	1.1371	0.9134	0.064*	
H6B	0.6289	1.0522	0.8571	0.064*	
H6C	0.6356	1.0139	0.9267	0.064*	

### Atomic displacement parameters (Å^2^)

**Table:**

	*U*^11^	*U*^22^	*U*^33^	*U*^12^	*U*^13^	*U*^23^
Cu1	0.0191 (4)	0.0213 (4)	0.0307 (4)	0.0111 (4)	0.0002 (3)	0.0007 (3)
V1	0.0197 (6)	0.0197 (6)	0.0226 (9)	0.0098 (3)	0.000	0.000
S1	0.0180 (8)	0.0194 (8)	0.0234 (7)	0.0099 (7)	−0.0012 (6)	0.0018 (6)
N1	0.027 (3)	0.030 (3)	0.020 (2)	0.016 (3)	−0.002 (2)	−0.003 (2)
N2	0.026 (3)	0.030 (3)	0.063 (4)	0.011 (3)	0.012 (3)	−0.007 (3)
C2	0.025 (4)	0.034 (4)	0.030 (3)	0.022 (3)	0.006 (3)	−0.002 (3)
O1	0.030 (2)	0.030 (2)	0.016 (3)	0.0150 (12)	0.000	0.000
C1	0.024 (4)	0.023 (3)	0.021 (3)	0.012 (3)	−0.002 (3)	−0.003 (3)
C4	0.022 (4)	0.029 (4)	0.039 (4)	0.010 (3)	−0.003 (3)	−0.003 (3)
C5	0.031 (4)	0.021 (4)	0.037 (4)	0.013 (3)	0.004 (3)	−0.004 (3)
C3	0.024 (4)	0.037 (4)	0.022 (3)	0.020 (3)	−0.001 (3)	0.003 (3)
C6	0.029 (4)	0.066 (5)	0.040 (4)	0.030 (4)	0.006 (3)	0.006 (4)

### Geometric parameters (Å, °)

**Table:**

Cu1—N1	2.033 (4)	N1—C5	1.344 (7)
Cu1—S1^i^	2.2886 (15)	N1—C1	1.344 (6)
Cu1—S1^ii^	2.3353 (15)	N2—C1	1.350 (7)
Cu1—S1	2.4701 (16)	N2—H2A	0.8600
Cu1—V1	2.6932 (11)	N2—H2B	0.8600
Cu1—Cu1^ii^	2.7725 (10)	C2—C3	1.366 (8)
Cu1—Cu1^i^	2.7725 (10)	C2—C1	1.387 (7)
V1—O1	1.618 (6)	C2—H2C	0.9300
V1—S1^ii^	2.2382 (15)	C4—C3	1.373 (8)
V1—S1^iii^	2.2382 (15)	C4—C5	1.374 (7)
V1—S1^i^	2.2382 (15)	C4—H4A	0.9300
V1—Cu1^iv^	2.6932 (11)	C5—H5A	0.9300
V1—Cu1^v^	2.6932 (11)	C3—C6	1.498 (7)
S1—V1^iii^	2.2382 (15)	C6—H6A	0.9600
S1—Cu1^ii^	2.2886 (15)	C6—H6B	0.9600
S1—Cu1^i^	2.3353 (15)	C6—H6C	0.9600
			
N1—Cu1—S1^i^	121.11 (14)	S1^iii^—V1—Cu1	126.41 (7)
N1—Cu1—S1^ii^	107.71 (14)	S1^i^—V1—Cu1	54.36 (4)
S1^i^—Cu1—S1^ii^	104.91 (7)	Cu1^iv^—V1—Cu1	90.91 (4)
N1—Cu1—S1	104.52 (13)	Cu1^v^—V1—Cu1	90.91 (4)
S1^i^—Cu1—S1	109.83 (6)	V1^iii^—S1—Cu1^ii^	73.01 (5)
S1^ii^—Cu1—S1	108.29 (5)	V1^iii^—S1—Cu1^i^	72.12 (5)
N1—Cu1—V1	132.40 (12)	Cu1^ii^—S1—Cu1^i^	112.24 (6)
S1^i^—Cu1—V1	52.63 (4)	V1^iii^—S1—Cu1	111.28 (7)
S1^ii^—Cu1—V1	52.27 (4)	Cu1^ii^—S1—Cu1	71.15 (4)
S1—Cu1—V1	122.30 (5)	Cu1^i^—S1—Cu1	70.41 (4)
N1—Cu1—Cu1^ii^	114.62 (14)	C5—N1—C1	116.4 (5)
S1^i^—Cu1—Cu1^ii^	124.25 (4)	C5—N1—Cu1	115.9 (4)
S1^ii^—Cu1—Cu1^ii^	57.07 (4)	C1—N1—Cu1	127.6 (4)
S1—Cu1—Cu1^ii^	51.37 (4)	C1—N2—H2A	120.0
V1—Cu1—Cu1^ii^	90.71 (3)	C1—N2—H2B	120.0
N1—Cu1—Cu1^i^	127.78 (13)	H2A—N2—H2B	120.0
S1^i^—Cu1—Cu1^i^	57.48 (4)	C3—C2—C1	121.3 (5)
S1^ii^—Cu1—Cu1^i^	123.48 (4)	C3—C2—H2C	119.4
S1—Cu1—Cu1^i^	52.52 (4)	C1—C2—H2C	119.4
V1—Cu1—Cu1^i^	90.71 (3)	N1—C1—N2	118.1 (5)
Cu1^ii^—Cu1—Cu1^i^	87.63 (4)	N1—C1—C2	121.6 (5)
O1—V1—S1^ii^	108.97 (5)	N2—C1—C2	120.2 (5)
O1—V1—S1^iii^	108.97 (5)	C3—C4—C5	119.3 (5)
S1^ii^—V1—S1^iii^	109.97 (5)	C3—C4—H4A	120.3
O1—V1—S1^i^	108.97 (5)	C5—C4—H4A	120.3
S1^ii^—V1—S1^i^	109.97 (5)	N1—C5—C4	124.1 (5)
S1^iii^—V1—S1^i^	109.97 (5)	N1—C5—H5A	117.9
O1—V1—Cu1^iv^	124.62 (3)	C4—C5—H5A	117.9
S1^ii^—V1—Cu1^iv^	54.36 (4)	C2—C3—C4	117.2 (5)
S1^iii^—V1—Cu1^iv^	55.61 (4)	C2—C3—C6	121.0 (5)
S1^i^—V1—Cu1^iv^	126.41 (7)	C4—C3—C6	121.8 (6)
O1—V1—Cu1^v^	124.62 (3)	C3—C6—H6A	109.5
S1^ii^—V1—Cu1^v^	126.41 (7)	C3—C6—H6B	109.5
S1^iii^—V1—Cu1^v^	54.36 (4)	H6A—C6—H6B	109.5
S1^i^—V1—Cu1^v^	55.61 (4)	C3—C6—H6C	109.5
Cu1^iv^—V1—Cu1^v^	90.91 (4)	H6A—C6—H6C	109.5
O1—V1—Cu1	124.62 (3)	H6B—C6—H6C	109.5
S1^ii^—V1—Cu1	55.61 (4)		

Symmetry codes: (i) *y*−1, −*x*+*y*, −*z*+2; (ii) *x*−*y*+1, *x*+1, −*z*+2; (iii) −*x*, −*y*+2, −*z*+2; (iv) −*y*+1, *x*−*y*+2, *z*; (v) −*x*+*y*−1, −*x*+1, *z*.
